# Fabrication of Water Jet Resistant and Thermally Stable Superhydrophobic Surfaces by Spray Coating of Candle Soot Dispersion

**DOI:** 10.1038/s41598-017-06753-4

**Published:** 2017-08-08

**Authors:** Talal F. Qahtan, Mohammed A. Gondal, Ibrahim O. Alade, Mohammed A. Dastageer

**Affiliations:** Laser Research Group, Physics Department & Center of Excellence in Nanotechnology, King Fahd University of Petroleum & Minerals, Dhahran, 31261 Saudi Arabia

## Abstract

A facile synthesis method for highly stable carbon nanoparticle (CNP) dispersion in acetone by incomplete combustion of paraffin candle flame is presented. The synthesized CNP dispersion is the mixture of graphitic and amorphous carbon nanoparticles of the size range of 20–50 nm and manifested the mesoporosity with an average pore size of 7 nm and a BET surface area of 366 m^2^g^−1^. As an application of this material, the carbon nanoparticle dispersion was spray coated (spray-based coating) on a glass surface to fabricate superhydrophobic (water contact angle > 150° and sliding angle < 10 °) surfaces. The spray coated surfaces were found to exhibit much improved water jet resistance and thermal stability up to 400 °C compared to the surfaces fabricated from direct candle flame soot deposition (candle-based coating). This study proved that water jet resistant and thermally stable superhydrophobic surfaces can be easily fabricated by simple spray coating of CNP dispersion gathered from incomplete combustion of paraffin candle flame and this technique can be used for different applications with the potential for the large scale fabrication.

## Introduction

Over the decades, carbon in nano-scale has been synthesized in various morphological structures, such as nanotube, nanowire, nanofiber, fullerenes and other carbonaceous nanomaterial. These materials have been used as sorbents^3^ antimicrobial agents^4^, environmental sensors^5^ and for the storage of gases^6, 7^. All these progress in the studies and applications of carbon nanomaterials are possible due to their novel physical, chemical and mechanical properties which arise from their morphology, high surface area, biocompatibility, low toxicity and chemical inertness inherent to their various nanostructures^8^. To realize the full potential application of carbon nanomaterials, systematic studies of carbon nanoparticle is essential in addition to other nanostructures^1, 2, 4, 9–11^. On this basis, active research on development of carbon nanoparticles (CNPs) possessing improved chemical and physical properties with potential for many technological applications is an ongoing effort^9–11^. Recent studies have demonstrated that CNPs can be a good material for photonic applications as they exhibit non- blinking bright fluorescence, emission spectra with tunable excitation, high photo-stability and good electroluminescence^11, 12^. Moreover, CNPs have shown better biocompatibility, steady fluorescence, chemical inertness and less toxicity compared to conventional heavy metal based quantum dots^11^. All these positive attributes of CNPs can be harnessed for its vital applications like cell imaging and optoelectronics^10, 13–16^.

Conventional top-down approaches adopted for the synthesis of CNPs involve disintegration of bulky carbon material using high impact mechanical process. However, these techniques naturally need high material cost, huge instrumentation and harsh operation conditions^17, 18^. Other possible alternative is the less cumbersome and cost effective bottom-up synthesis route, where CNPs are synthesized by combustion and/or chemical process using readily available organic molecular precursors^19, 20^.

In the present work, highly stable carbon nanoparticle (CNP) dispersion was synthesized from candle soot obtained through incomplete combustion of paraffin candle flame. The synthesized CNP dispersions were found to be highly stable in different solvents (acetone, isopropanol and ethanol) for more than a year under ambient conditions. Superhydrophobic surfaces (water contact angle >150° and sliding angle <10°) were fabricated by simply spray coating the candle soot dispersion on a glass surface using a spray-gun (spray-based coating). The superhydrophobic surfaces thus fabricated exhibit superior resistance to drop impact, water jet impingement and are thermally stable, compared to the same surfaces fabricated from direct candle flame soot deposition (candle-based coating). The striking feature of this method of synthesis is that the experimental set up is simple and the raw material is inexpensive. Moreover, the synthesized dispersion can be easily applied for large-scale fabrication of thermally stable superhydrophobic surfaces with high resistance to drop impact and water jet impingement.

## Results and Discussions

### Characterization of Candle Soot Dispersion and Coated Surfaces

**Figure 1:**
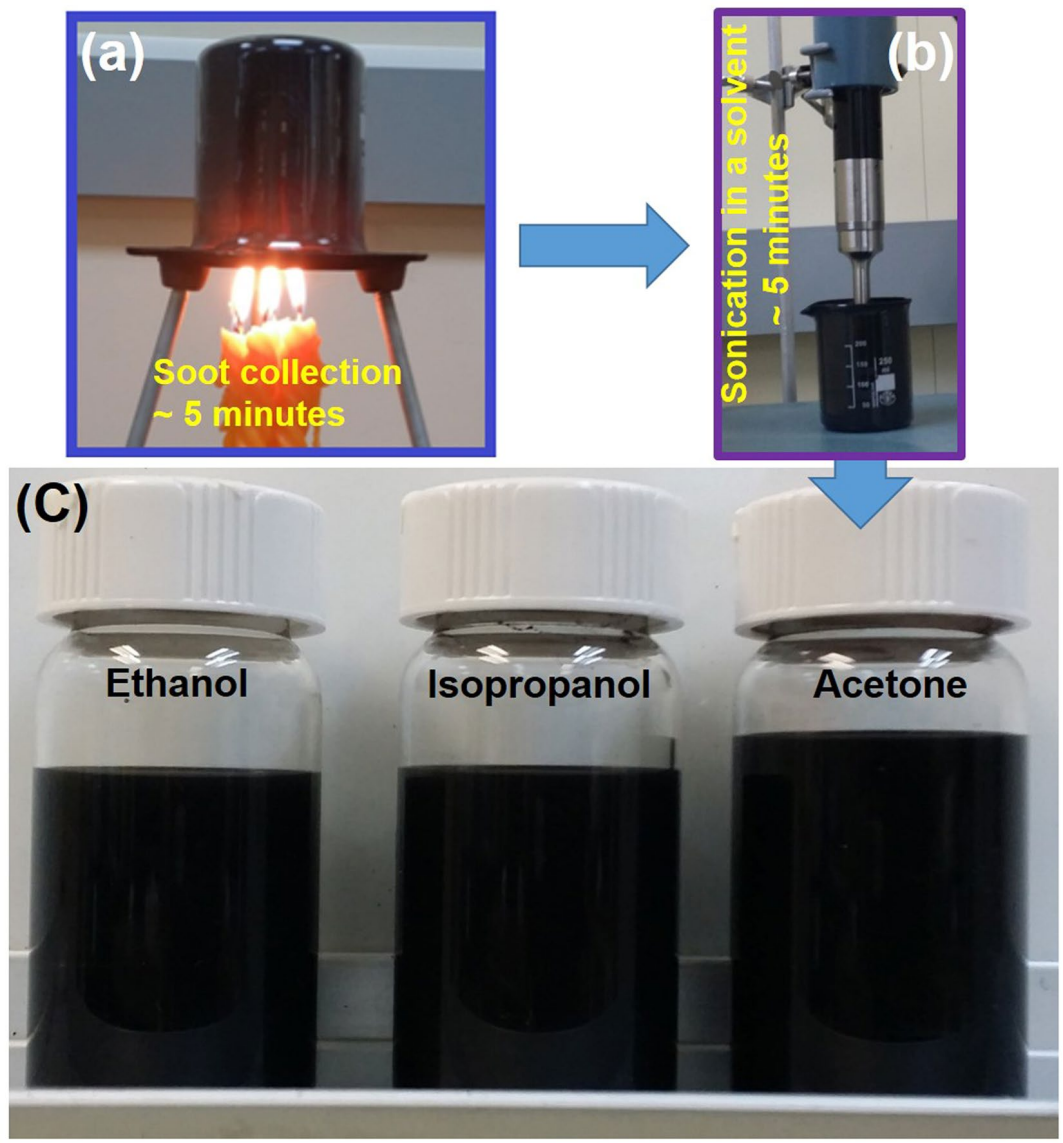
Preparation of candle soot dispersions. (**a**) Candle soot collection: A 250 ml glass beaker is inverted on 5 candle flames such that the zone of incomplete combustion strikes the walls of the beaker for 5 minutes (~250 mg of soot). (**b**) Sonication of collected candle soot in different solvents: The highly stable, homogeneous dispersion of the candle soot in different solvents were prepared by simply mixing the candle soot coated on the beaker with the solvents (250 mg soot dispersed in 250 ml of each solvent), and sonicating it for 5 minutes (**c**) Photograph of prepared candle soot nanoparticles in different solvent as indicated (ethanol, isopropanol and acetone).

**Figure 2:**
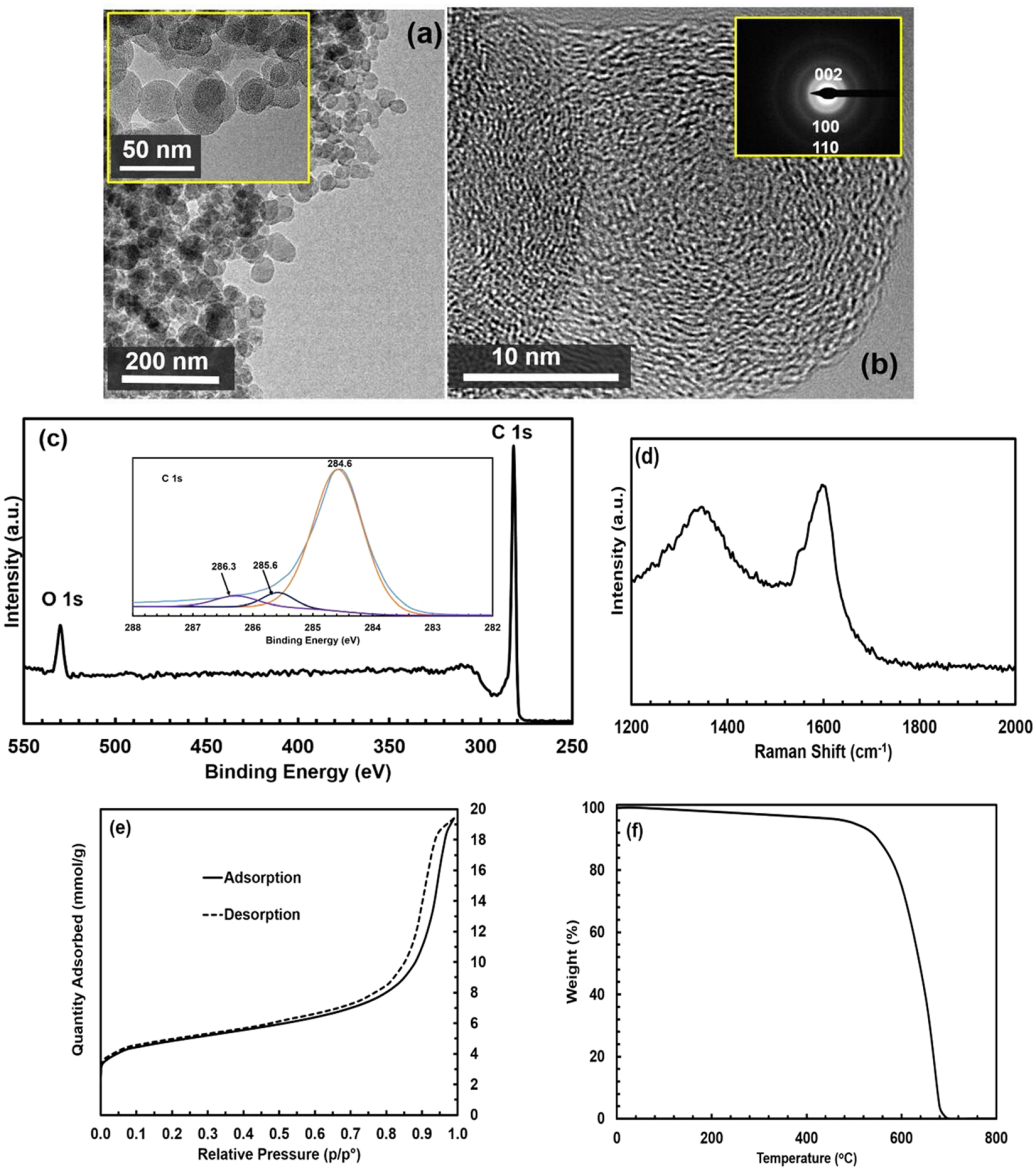
Characterization of the prepared Candle soot. (**a**) TEM images with higher magnification (inset): The candle soot dispersion consists of a large number of nanospheres in the size range of 20 to 50 nm (**b**) High resolution TEM image of the candle soot and its SAED pattern (inset). It indicates the presence of few pieces of crystalline carbon nanospheres in candle soot along with amorphous carbon, which is further confirmed by Selected Area Electron (**c**) XPS spectrum. It indicates that the candle soot consists mostly of carbon nanoparticles. The inset in the figure shows the deconvolution of C1s XPS peak (**d**) Raman shift of the candle soot. The peak at 1350 cm^−1^ (D bands) accounts for the amorphous carbon and the one at 1580 cm^−1^ (G bands) corresponds to E_2g_ mode of graphite due to the vibration of sp^2^-bonded carbon atoms in a 2D hexagonal lattice (**e**) Surface Area analysis of the candle soot. It is mesoporous material with an average pore size of 7 nm and a BET surface area of 366 m^2^g^−1^. (**f**) Thermogravimetric analysis of the candle soot, which shows the weight loss of the candle soot, is less than 3.5% up to the temperature of 450 °C.

The preparation procedure for the candle soot dispersion is shown in Fig. 1. The TEM images of the dispersion of carbon nanoparticles (CNPs), through incomplete combustion of paraffin candle flame are shown in Fig. 2a, and b. It is quite clear from Fig. 2a that the candle soot dispersion consists of a large number of nanospheres in the size range of 20 to 50 nm and the magnified version of the TEM image presented in the inset of Fig. 2a shows that the carbon nanospheres are formed from the irregularly patterned spherical carbon nanoparticles linked through a weak Van-der-Waals interaction. The agglomeration of carbon nanosphere results due to the presence of dangling bonds, which makes the surface highly reactive^18, 21, 22^, Fig. 2b indicates the presence of few pieces of crystalline carbon nanospheres in candle soot along with amorphous carbon, which is further confirmed by Selected Area Electron Diffraction pattern (SAED) shown in the inset of Fig. 2b. The observed diffraction rings in SAED pattern are due to (002), (100) and (110) planes of crystalline candle soot nanospheres^23, 24^.

The chemical composition of the paraffin candle soot was examined by EDS and XPS. The EDS measurements reveal that the main component is carbon (Atomic percentage − 97.0%) and the minor component is oxygen (Atomic percentage − 3.0%). The presence of strong C1s peak in XPS analysis depicted in Fig. 2c indicates that this candle soot consists mostly of carbon (Atomic percentage of C1s and O1s are 97.2 and 2.8 respectively) and are free from other elements of organic contaminates, originating from paraffin and/or the environment, which is in a good agreement with EDS results. The inset of Fig. 2d shows that C1s is decomposed into three peaks centered at 284.6 eV, 285.6 eV and 286.3 eV which corresponds to sp^2^ hybridized C = C, sp^3^ hybridized C-C and C-O bonds respectively^25^. The presence of amorphous and graphitic carbon in the candle soot is further substantiated with the Raman spectrum, shown in Fig. 2d, where the peak at 1350 cm^−1^ (D bands) accounts for the amorphous carbon and the one at 1580 cm^−1^ (G bands) corresponds to E_2g_ mode of graphite due to the vibration of sp^2^-bonded carbon atoms in a 2D hexagonal lattice. The presence of the G band indicates that the candle soot consist of highly ordered pyrolytic graphite^18^.

The N_2_ adsorption-desorption isotherm of the candle soot is given in Fig. 2e, which resembles the Type IV isotherm described by Brunauer *et al*.^14^, where the increase in absorption volume at high relative pressure indicates the presence of majority amount of mesopores and negligibly small amount of micropores, however; the presence of hysteresis loop at high relative pressure rules out the presence of macrospores in the candle soot. The textural parameters obtained quantitatively shows that candle soot has an average pore size of 7 nm and large BET surface area of 366 m^2^g^−1^.

In order to study the thermal stability of candle soot, thermal gravimetric analysis (TGA) was carried out and it is clear from Fig. 2f that up to the temperature of 450 °C, the weight loss of the candle soot is less than 3.5%, which indicates the absence of significant decomposition. This result ensures that the candle soot dispersion can be used for any applications that require the functional temperature below 450 °C.

### Application of the candle soot dispersion to fabricate superhydrophobic surfaces

Water repellent superhydrophobic surfaces have found its place in many vital applications, such as oil-water separation^26, 27^, corrosion prevention^28, 29^, self-cleaning glass windows^30^ and windscreens designs^31^ and hence received tremendous attention both in academia and industry. Superhydrophobicity of a surface is characterized by the high water contact angle (above 150°) and low sliding angle (below 10°)^32–34^, and the two underlying factors that results in the realization of such surfaces are low surface energy and micron-to-nanoscale hierarchical surface roughness^34–36^. Many deposition methods such as sol-gel processing^37^, chemical vapor deposition^36^, laser ablation^38, 39^ and radio frequency plasma etching^40^ have been used for the fabrication of superhydrophobic surfaces. While these approaches have yielded the expected results, their complexity, huge power requirement and cost associated with the materials present a main challenge for the fabrication of low-cost superhydrophobic surfaces on a large scale. Candle soot, an inexpensive carbon material with promising morphology has been demonstrated as a material suitable for fabrication of superhydrophobic surfaces. However, further modification is required before it can be suitably used for fabrication superhydrophobic surfaces because the particle-particle interaction in the candle soot is weak^41–46^. To this end, Seo *et al*.^21^ prepared a superhydrophobic surface from candle soot using paraffin wax as binder to strengthen the interactions between the soot particles. Bayer *et al*.^45^ synthesized durable superhydrophobic surfaces including flexible substrates using flame from a butane burner in conjunction with triboelectric particle deposition process. In addition, Esmeryan *et al*.,^46^ used soot from rapeseed oil in combination with a specially-designed cone-shaped aluminum chimney to achieve superhydrophobic surface without the use of further chemical treatment. So far, significant progress has been made in terms of simplified fabrication technique for soot-based superhydrophobic surfaces. The interest to further explore novel, simple and inexpensive method for fabricating superhydrophobic surfaces is still very attractive.

**Figure 3:**
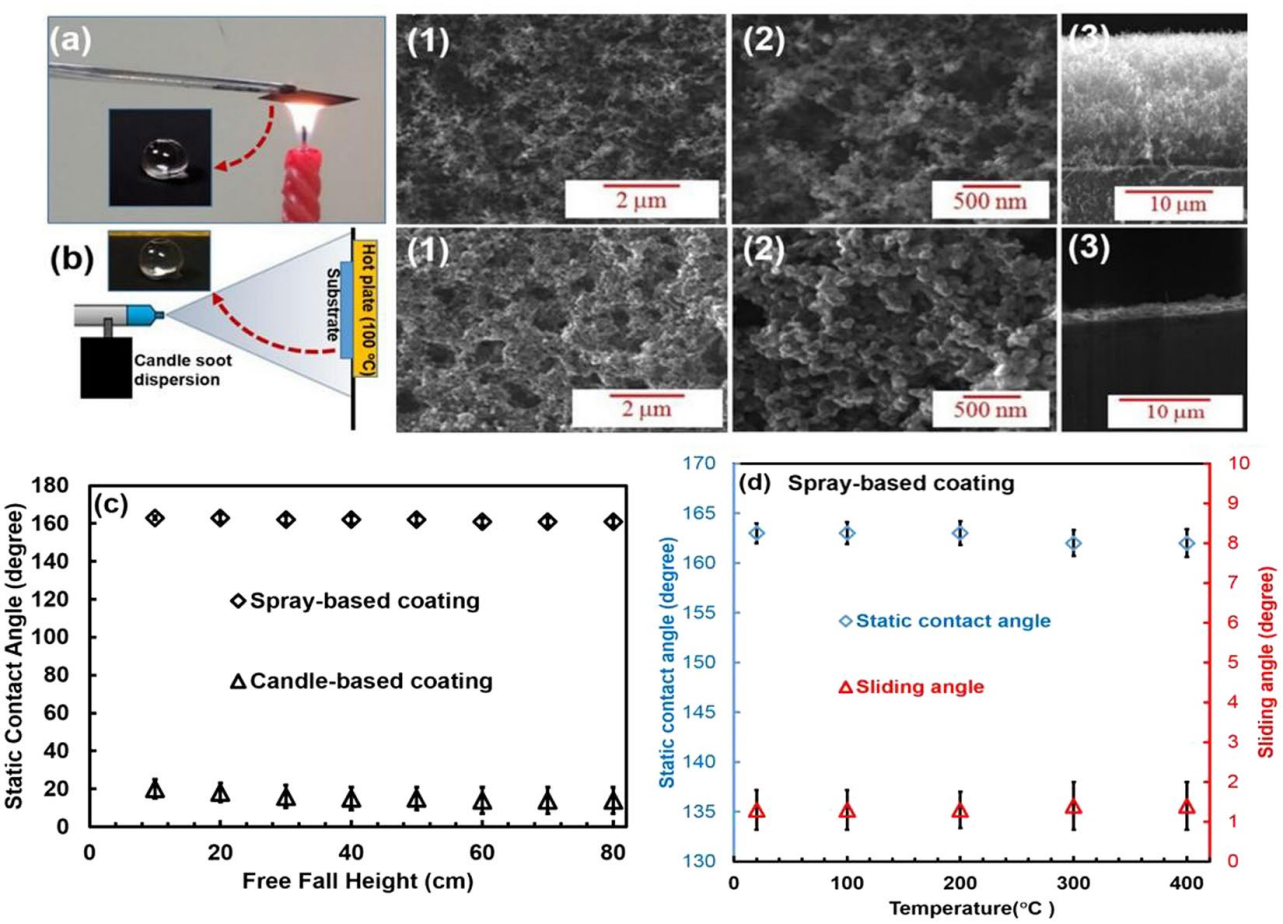
Fabrication of superhydrophobic surfaces using candle soot dispersions. (**a**) Direct candle flame soot deposition. (a-1 and 2) FESEM images of the surfaces prepared by direct candle flame soot deposition with different magnifications, (a-3) Cross sectional view of the surfaces prepared by direct candle flame soot deposition. (**b**) Spray coating method. (b-1 and 2) FESEM images of spray based coatings with different magnifications and (b-3) cross sectional view of spray based coatings. It shows the compact nature of this coating. (**c**) Robustness test of both surfaces. (**d**) Thermal stability test of spray-based coating. Both surfaces (candle-based coating and spray-based coating) are superhydrophobic with high static water contact angle of 163^o^ ± 2 and very low sliding contact angle less than 2^o^. The surfaces fabricated by the candle-based coating lose its superhydrophobicity by the impact of water droplets, whereas the spray-based surface is very robust under the same test.

In this work, a low cost facile approach for the fabrication of stable superhydrophobic surfaces by simple spraying of candle soot dispersion on the surfaces was adopted. The surface morphologies, wettability, resistance to drop impact/water jet impingement and thermal stability of the superhydrophobic surfaces, fabricated by the spray coating method is illustrated in Fig. 3 by comparing these characteristics for the superhydrophobic surfaces fabricated by direct candle flame soot deposition. From the contact angle snapshots shown in insets of Fig. 3-a and Fig. 3-b, it is quite clear that the surfaces fabricated by both the techniques (direct candle flame soot deposition and spray coating) are superhydrophobic with high static water contact angle of 163° ± 2 and very low sliding contact angle, which is less than 2°. Although, the candle based coating is more suitable to coat all surfaces including polymeric substrate, the surfaces fabricated by this method lose its superhydrophobicity by the impact of water droplets due to the fragile interaction between the surface and carbon nanoparticles^21, 41^.

Figures 3-a 1 and 2 and Figs. 3-b 1 and 2 show the FE-SEM images of superhydrophobic surfaces fabricated by direct candle flame soot deposition and spray coating respectively, where we can notice that both surfaces reveal the patterns of irregular nanoparticle network with micron-to-nanoscale surface roughness, the condition necessary for superhydrophobicity. However, it is clear from the cross-sectional views of SEM images shown in Fig. 3a-3 and Fig. 3b-3, the thicknesses of surfaces by direct candle flame soot deposition and spray coating-based method are ~15 µm and ~ 3 µm respectively and also the spray coating-based surface shows hierarchical and compact nature.

The drop impact resistance of the surface fabricated by direct candle flame soot deposition and spray coating methods were tested by observing its ability to retain static contact angles after striking the surfaces by water droplets from a certain height and these results are depicted in Fig. 3c. When 1000 drops of water were allowed to fall on the surfaces from varying heights from 10 cm to 80 cm, the static contact angle of the surface fabricated by direct candle flame soot deposition dropped from 163° to 20° when the water drops fell from the height as low as 10 cm, indicating that the surface lost its superhydrophobicity, whereas in the case of the spray coated surfaces, the static contact angle remained intact at 163° even when the water drops fell from the height of 80 cm. This clearly proves the outstanding resistance to drop impact of superhydrophobic surfaces fabricated by spray coating-based method as compare to one made by direct candle flame soot deposition. The water jet resistance of the spray coating-based surface is demonstrated in supplementary videos S1 and S2, where we can notice that superhydrophobic surface fabricated by direct candle flame soot deposition was destroyed on application of water jet, while the spray coating-based surface clearly endures the impacts and still exhibit superhydrophobic properties under same stream of water jet. This observation can be attributed to the higher compactness of the spray coating-based surfaces as revealed from the SEM studies.

The thermal stability of the spray coating-based surface was tested by examining its ability to retain its superhydrophobicity after annealing the surface in the temperature range of 100–400 °C. It is quite clear from Fig. 3-d that both the static contact angle and the sliding angles remain at their initial values even after annealing the surface at a temperature of 400 °C, indicating that the superhydrophobicity is intact and the surface is thermally stable at a temperature as high as 400^o^C. The thermal stability of the spray coating-based surface was further confirmed with thermogravimetric studies as described earlier. All these studies testify that the spray coating -based surface shows better resistance to drop impact/ water jet impingement and thermal stability than superhydrophobic surfaces fabricated by direct candle flame soot deposition.

## Conclusions

In this work, we used a cheap and easily available paraffin candle to prepare highly stable carbon nanoparticles (20–50 nm) dispersions in acetone. The candle soot is mesoporous nanomaterial with an average pore size of 7 nm and large BET surface area of about 366 m^2^g^−1^, which can be used for different applications. To show the advantage of our nanomaterial for practical and large-scale applications, the synthesized carbon nanomaterial was used to fabricate superhydrophobic surfaces using spray coating-based technique. The spray coating-based surface is thermally stable over a wide temperature range up to 400 ^o^C and exhibits superior resistance to drop impact, water jet impingement, compared to the surface fabricated by direct candle flame soot deposition. The results of this work assures that the potential of this technique can be used for the fabrication of mesoporous carbon nanomaterials that can be used for large-scale practical applications, that require thermally stable robust superhydrophobic surfaces for the solar cell electrodes and band gap engineering of metal oxides.

## Experimental Procedures

The paraffin candles used in this work are the ones commercially available in the local market and all the solvents were of analytical grade from Sigma Aldrich. A 250 ml glass beaker was inverted on 5 candle flames such that the zone of incomplete combustion strikes the wall of the beaker for 5 minutes, gathering approximately 250 mg of the candle soot. Highly stable homogeneous dispersions of the candle soot in three solvents were prepared by simply mixing 250 mg of candle soot in 250 ml, 500 ml and 750 ml of three solvents (acetone, isopropanol and ethanol) followed by sonication for 5 minutes. For the preparation of superhydrophobic surfaces, 10 ml of all the above nine different combinations of candle soot dispersions were spray coated on 4 cm^2^ glass substrates using a spray gun (McMaster Carr). It was found that 10 ml of 250 mg in 250 ml dispersion in all three solvents is the optimum quantity and concentration needed to cover a 4 cm^2^ substrate by soot to fabricate superhydrophobic surfaces. Although, 10 ml of 250 mg in 250 ml dispersion in all three solvents gave same result, we chose acetone as a solvent to fabricate superhydrophobic surfaces in this study. The spray gun was operated with nitrogen gas at pressure of 170 kPa. In order to make the solvent evaporate readily and get uniform spray-based superhydrophobic surfaces, the substrates were fixed on an electric heater maintained at 100 ^o^C during the spray coating process. To study the relative merit of the spray-based superhydrophobic surfaces, we fabricated candle-based superhydrophobic surfaces by direct candle flame soot deposition also, where the candle flame was directly intercepted by the substrate. These two variants of surfaces were used for the studies of wettability, stability against drop impact, water jet impingement and thermal stability.

X-ray Photoelectrons Spectroscopy (XPS), Energy-dispersive X-ray spectroscopy (EDS) and Selected Area Electron Diffraction (SAED) techniques were used to study the chemical and phase composition of the candle soot. The particle size and the morphology of the candle soot were investigated using Transmission Electron Microscopy (TEM), whereas the same for the coated surfaces were carried out using Field Emission Scanning Electron Microscopy (FE-SEM). Raman spectrometer with an excitation wavelength of 514.5 nm was used to obtain the Raman spectrum of the candle soot. The BET surface area, pore size and pore volumes of the samples were measured on micromeritics Tristar surface area and porosimetry analyzer (Micrometrics, USA) using liquid N_2_ adsorption-desorption at −196 °C. Prior to BET measurements, the samples were degassed at 250 ^o^C for 3 hours to eliminate impurities and moisture. In addition to this, thermogravimetric analysis (TGA) was also used to investigate the thermal degradation of the candle soot.

A goniometer was used for the static contact and sliding angle measurements of water drops (~ 10 µL) on the superhydrophobic surfaces. For studying the stability of the superhydrophobic surfaces against the drop impact, a count of thousand water drops were allowed to hit the coated substrates kept at 45° inclination from varying height, and subsequently, the static contact angle and the sliding angle as a function of water drop height were recorded. In order to study the thermal stability of the spray-based superhydrophobic surfaces, the surfaces were systematically annealed from 100°–400 °C for 2 hours thereafter the static contact and the sliding angles were measured on the annealed surfaces at room temperature.

## Electronic supplementary material

video 1

video 2

Supplementary Information
