# Supplementary material for: Fabrication of Water Jet Resistant and Thermally Stable Superhydrophobic Surfaces by Spray Coating of Candle Soot Dispersion
Source: Sci Rep. 2017 Aug 8;7:7531. doi: 10.1038/s41598-017-06753-4 (PMC5548922; doi:10.1038/s41598-017-06753-4)
Supplement: Supplementary file 3 — Supplementary Information [file 41598_2017_6753_MOESM3_ESM.pdf]

Fabrication of Water Jet Resistant and Thermally Stable Superhydrophobic Surfaces by Spray Coating of Candle Soot Dispersion

Talal F. Qahtan, Mohammed A. Gondal*, Ibrahim O. Alade, Mohammed A. Dastageer

Laser Research Group, Physics Department & Center of Excellence in Nanotechnology

Corresponding authors' email:* magondal@kfupm.edu.sa *(M.A. Gondal)***

Telephone: +9663-8602351/8603274; *Fax:* +9663-8604281

Legends of Videos:

Video S1: Testing of mechanical robustness of the surface coated with direct candle flame soot deposition, in which the surface is destroyed on application of water jet.

Video S2: Testing of mechanical robustness of the surface coated with spray coating-based technique, in which the surface clearly endures the impacts and still exhibit superhydrophobic properties under the stream of water jet.
